# Enhancing Fatigue Resistance of Polylactic Acid through Natural Reinforcement in Material Extrusion

**DOI:** 10.3390/polym16172422

**Published:** 2024-08-27

**Authors:** Carolina Bermudo Gamboa, Sergio Martín-Béjar, Fermín Bañón García, Lorenzo Sevilla Hurtado

**Affiliations:** Department of Civil, Materials and Manufacturing Engineering, Engineering School, University of Malaga, 29071 Malaga, Spain; fermin.banon@uma.es (F.B.G.); lsevilla@uma.es (L.S.H.)

**Keywords:** Material Extrusion, polylactic acid, natural fibers, fatigue behavior, dimensional accuracy

## Abstract

This research paper aims to enhance the fatigue resistance of polylactic acid (PLA) in Material Extrusion (ME) by incorporating natural reinforcement, focusing on rotational bending fatigue. The study investigates the fatigue behavior of PLA in ME, using various natural fibers such as cellulose, coffee, and flax as potential reinforcements. It explores the optimization of printing parameters to address challenges like warping and shrinkage, which can affect dimensional accuracy and fatigue performance, particularly under the rotational bending conditions analyzed. Cellulose emerges as the most promising natural fiber reinforcement for PLA in ME, exhibiting superior resistance to warping and shrinkage. It also demonstrates minimal geometrical deviations, enabling the production of components with tighter dimensional tolerances. Additionally, the study highlights the significant influence of natural fiber reinforcement on the dimensional deviations and rotational fatigue behavior of printed components. The fatigue resistance of PLA was significantly improved with natural fiber reinforcements. Specifically, PLA reinforced with cellulose showed an increase in fatigue life, achieving up to 13.7 MPa stress at 70,000 cycles compared to unreinforced PLA. PLA with coffee and flax fibers also demonstrated enhanced performance, with stress values reaching 13.6 MPa and 13.5 MPa, respectively, at similar cycle counts. These results suggest that natural fiber reinforcements can effectively improve the fatigue resistance and dimensional stability of PLA components produced by ME. This paper contributes to the advancement of additive manufacturing by introducing natural fiber reinforcement as a sustainable solution to enhance PLA performance under rotational bending fatigue conditions. It offers insights into the comparative effectiveness of natural fibers and synthetic counterparts, particularly emphasizing the superior performance of cellulose.

## 1. Introduction

Additive manufacturing is a growing technology with broad applicability across multiple industries. One of the most established additive manufacturing technologies is ME (Material Extrusion). This technology is characterized by its ability to work with a wide range of materials [1,2,3].

The versatility of ME technology has allowed its use in many industries. It can be used in industries with less-demanding tolerances and requirements to advanced sectors such as automotive, medical, and aerospace [1,4,5,6,7,8,9].

One of the most widely used materials for ME applications is PLA (polylactic acid), which has been extensively researched. However, pure PLA has limitations in terms of its mechanical properties [10]. This makes it less suitable for advanced sectors such as aerospace, where materials with more demanding properties are required [11].

Combining PLA with continuous carbon fiber reinforcements has been stated to be an effective strategy for improving the mechanical properties of the material [3]. Carbon fiber is known for its high strength and stiffness, making it an excellent reinforcement for PLA [12,13]. Pertuz et al. [14] found that the isotropic carbon fiber layers had the highest tensile strength at 165 MPa. Incorporating carbon fibers into PLA increases the tensile strength, stiffness, and impact resistance of the resulting material. This is supported by the results of Saharudin et al. [15] who compared the mechanical properties of PLA and PLA + continuous carbon fiber in ME technology. The authors found that the addition of carbon fibers had a significant effect on the strength of the models produced, increasing both tensile strength and Young’s modulus. This makes it more suitable for applications in demanding sectors such as aerospace, where high structural performance is required. The review by Najmon et al. [16] showed the interest in polymer additive manufacturing techniques, highlighting the use of ME parts in NASA’s Mars Rover.

In addition, the focus on sustainability and environmental impact has led to an emphasis on recycling in the manufacturing process of CFRP (carbon fiber-reinforced composite) components [17]. The manufacturing of CFRPs produces defective or scrap parts that would normally be considered waste. However, these parts can be processed to separate the carbon fiber reinforcement from the thermoset matrix. The recovered carbon fiber reinforcement can be combined with the thermoplastic PLA to form a new material with improved properties [18]. This recycling approach not only reduces the amount of waste generated but also takes advantage of the mechanical performance benefits of carbon fiber [19].

Other reinforcement options for PLA, such as natural fibers derived from waste from other industries, are being explored in addition to carbon fiber reinforcement [20,21]. These natural fibers, such as hemp, flax, or cellulose, can be used as reinforcements in combination with PLA [22,23]. By reusing this waste in the form of natural fibers, there are environmental and economic advantages [24]. It reduces the amount of waste going to landfills and makes use of an abundantly available material. Combining these natural fibers with PLA improves the strength and stiffness of the final material, making it suitable for applications that require better mechanical properties. In their research, Rajendran et al. [25] indicated that baste fibers with 50–90% cellulose crystallinity are used in various automotive components to achieve better mechanical properties of the products in terms of modulus, strength, and stiffness.

Thus, combining PLA with carbon fiber reinforcements and natural fibers offers a promising solution to overcome the mechanical property limitations of pure PLA. These strategies not only improve material performance but also contribute to sustainability by using recycled materials and reducing waste [20,26,27,28].

In the field of additive manufacturing of polymers such as PLA, there is still no specific standard for the characterization of mechanical properties. In the absence of a specific standard, the tests used for the mechanical characterization of metallic alloys are usually adopted and adapted to these polymeric materials [29]. Tensile tests are among the most commonly used mechanical tests to characterize the behavior of the PLA.

An aspect of great interest to sectors such as aeronautics or the automotive industry, however, is the study of the fatigue life of these materials, especially when combined with reinforcements, whether carbon or natural fibers [14]. The study of how materials behave when subjected to repeated loading and unloading cycles may be relevant in applications where the material is expected to withstand dynamic loads over a long period of time [30]. In their research, Fischer et al. [31] investigated the behavior of ME parts using fatigue tests. The authors showed that ME anisotropy affects the durability of the specimens at higher loads, while the S–N curves converge at lower loads for different orientations. In addition, chemical smoothing improves the tensile strength and reduces the surface roughness of the specimens in the vertical direction. Lee et al. [32] studied the effects of fatigue on acrylonitrile butadiene styrene (ABS) materials obtained by ME. The authors tested a bone-like geometry based on UNE EN ISO 527-1 [33]. These tests provide information on the strength and deformation properties of the material under axial loading [34].

In the specific case of the fatigue life of polymers produced by additive manufacturing techniques and in combination with reinforcements, there is a gap in the scientific literature with regard to rotational fatigue testing [14,30]. These tests involve the application of cyclic rotating loads to the specimens and are important in assessing the fatigue behavior of materials under real-life conditions [35,36].

Despite the extensive research on the fatigue behavior of 3D-printed materials, this study presents several key aspects that highlight its novelty and contribution to the field. Unlike previous studies that focus primarily on synthetic fibers or unreinforced polymers, our research specifically examines the rotational fatigue resistance of PLA reinforced with natural fibers such as cellulose, coffee, and flax. This approach not only enhances the mechanical properties of PLA but also aligns with sustainable and environmentally friendly practices, addressing a growing concern in material science [29]. While earlier researchers, such as Gomez-Gras et al. [30] and Fischer and Schöppner [31], investigated general fatigue testing of FDM materials, our study fills a significant gap by targeting rotational fatigue behavior, which is crucial for applications subjected to cyclic rotational stresses, such as those in the automotive and aerospace industries. Furthermore, our work evaluates the dimensional accuracy and geometric stability of 3D-printed components, correlating these factors with fatigue performance, an aspect less emphasized in prior studies [37,38]. By comparing the performance of natural fiber-reinforced PLA with both unreinforced PLA and PLA reinforced with carbon fibers [14], our research highlights the advantages and potential applications of using natural fibers. Additionally, we provide a comprehensive fractography analysis using high-quality SEM images, offering deeper insights into failure mechanisms and material improvement [35]. This focus on natural fiber reinforcements derived from waste products promotes the circular economy and reduces the environmental impact, setting our study apart in terms of sustainability [17]. Therefore, the novelty of our work lies in its specific focus on applying rotational bending fatigue testing to natural fiber-reinforced PLA, an approach rarely explored in existing studies that typically focus on tensile or compression fatigue. Additionally, our detailed evaluation of dimensional and geometric stability, combined with fatigue results, offers new insights into the relationship between 3D-printing parameters and the fatigue resistance of the specimens, providing valuable data for advanced engineering applications.

The lack of specific studies in this area limits the understanding of how the combination of PLA with carbon or natural reinforcements in additive manufacturing techniques affects the fatigue life of the resulting components. Further research and analyses are required to fully understand these aspects and provide relevant data for the design and application of 3D-printed components in demanding sectors such as the aerospace and automotive industries.

## 2. Materials and Methods

The experimental methodology employed in this study for investigating the fatigue behavior and dimensional deviations is outlined as follows.

### 2.1. Sample Manufacture and Dimensional and Geometrical Control

In this study, the ISO 1143:2021 standard [39] was chosen to define the specimen geometry and conduct the rotational bending fatigue tests. Currently, there is no specific standard for rotational bending fatigue testing of polymeric materials or polymeric composites. In the absence of a dedicated standard for polymers, it is common practice to adopt and adapt standards used for metallic materials. This standard has been successfully adapted in prior studies to evaluate the fatigue behavior of polymeric and composite materials [40,41]. This approach ensures consistency and comparability with previous research.

**Figure 1:**
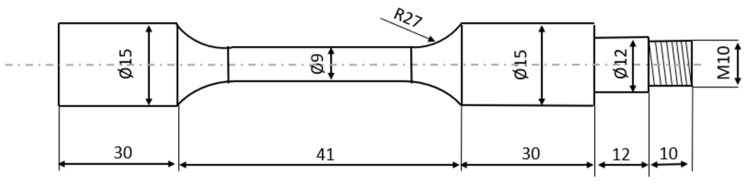
ISO 1143:2010 standard [39] sample (measured in mm).

Also, the geometry defined by the standard allows for the adaptation of specimens for use with specific fatigue testing equipment, ensuring reliable and repeatable measurements (Figure 1). The standardized approach minimizes variables that could arise from non-standardized specimen geometries, thereby improving the reliability of the findings.

**Figure 2:**
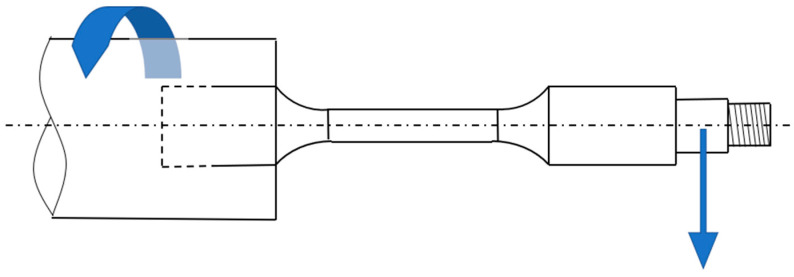
Rotational bending fatigue fixation.

In order to adapt the specimens to be tested with the fatigue testing equipment (as depicted in Figure 2), an extension is added to the end of the sample, providing the requisite dimensions for accommodating the load and the load-securing nut. These dimensions are 12 mm in diameter with a Ø12 mm end and 10 mm in diameter with a Ø10 mm end, respectively.

Regarding the materials subjected to testing, a variety of PLA composites containing natural fibers were chosen. This selection is presented in Table 1. Furthermore, the fatigue performance of these materials was juxtaposed with that of PLA, high-resistance (HR)-PLA, and PLA reinforced with carbon fiber (CF). In this initial study, ready-to-print composite filaments provided by the manufacturer were utilized. Consequently, the percentage of reinforcement fibers in the materials was predetermined by the supplier and is specified in Table 1. These contents were not varied as part of our experimental setup.

As discussed in the introduction, PLA stands out as an attractive choice for 3D printing due to its excellent printability and biodegradable nature. Typically, it requires lower printing temperatures and demonstrates resistance to warping effects. Nevertheless, its mechanical properties present a notable disadvantage. While printed PLA components may exhibit suboptimal mechanical characteristics, this material can be synergistically combined with other substances as reinforcement, with the aim of enhancing its mechanical performance. In light of the prioritization of PLA’s biodegradability and environmental sustainability, this study focuses on PLA reinforced with natural fibers, including cellulose, linen, and coffee, among others. In comparison to PLA combined with carbon fiber (PLA + CF), the entire printed structure is biodegradable, and industrial waste from other sectors can be repurposed to produce these printing filaments. Consequently, by concurrently improving mechanical properties and demonstrating environmental responsibility, these materials emerge as a favorable choice for 3D-printed components.

The specimens are fabricated using the Raise 3D E2 printer, an ME (Material Extrusion) technology system, with a working space of 330 × 240 × 240 mm. This printer features dual independent extruders, accommodates 1.75 mm filament, employs a 0.4 mm nozzle, reaches a maximum printing speed of 150 mm/s, and maintains a maximum printing temperature of 300 °C along with a maximum bed temperature of 110 °C. The specific printing parameters adopted for this study are outlined in Table 2. It is worth noting that the specimens were printed in a horizontal orientation. This orientation was selected due to the observed reduction in the fatigue life of specimens printed vertically (with layers parallel to the applied load), a phenomenon established in prior research [30,41].

The printing parameters chosen for this study, including the layer thickness, temperature, and speed, were based on prior research and experimentation. These parameters have consistently yielded reliable and reproducible results in our previous studies [40,42].

**Figure 3:**
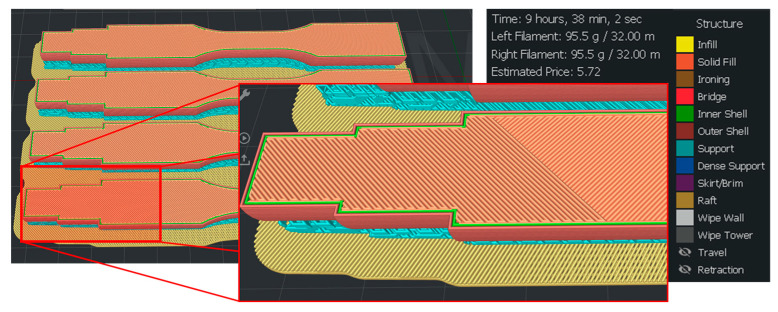
Samples sliced with the IdeaMaker software and interior filling detail.

The sample designs were created using SolidWorks (Dassault Systems [43]) and subsequently imported as .STL files into the 3D Slicer Software IdeaMaker [44], as depicted in Figure 3. In accordance with the ISO 12107:2012 standard [45], four samples were produced for each material, a measure taken to ensure test repeatability with a 95% confidence level and a 50% failure probability. The inclusion of a mirror-printing option allowed for the simultaneous fabrication of two separate materials. Figure 3 provides an overview of the distribution of the samples, along with their respective printing times, estimated durations, and a magnified view of the interior detailing the infill structure.

Moreover, a PLA control series was produced to enable the analysis and comparison of the performance of the composite materials with the polymer material in isolation.

**Figure 4:**
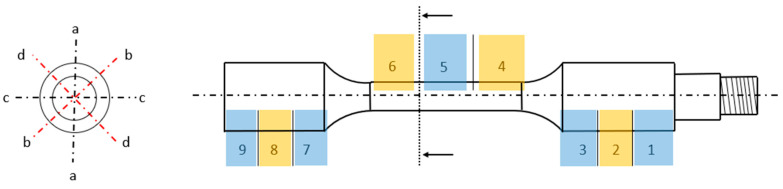
Dimensional measurement. Measurement areas (1–9) and measurement points at the same area (a–d), separated 45°.

With respect to dimensional deviations, the diameter of the printed components was assessed at various locations on the samples, as illustrated in Figure 4. While the central or calibrated sections (4, 5, and 6) represent the primary focus of the specimens, the extremities (1–3 and 7–9) are also examined. In each of these regions (1–9), four measurements were obtained at 45° intervals (a–d). The diameter was measured using a two-contact outside micrometer for exterior measurements (MITUTOYO, with a usable range of 0–25 mm and an error margin of E = 0.01 mm). This methodology was systematically applied to all printed samples to establish a correlation between the various composite materials and the observed dimensional deviations, ultimately aiding in the calculation of stress at the point of fatigue fracture.

Geometric deviations were assessed using a geometrical deviation measurement machine (ACCRETECH, model RONDCOM NEX) with a sensitivity of 0.01 µm. To initiate the process, the specimens were securely positioned on the machine’s level surface, and the zero position was configured. The parameters under scrutiny in this case study include roundness (RON), straightness (STR), and cylindricity (CYL). The geometrical deviation measurement machine captures values for RON and SRT, with CYL being subsequently calculated based on these measurements.

To ensure the consistency and reliability of measurements, multiple readings were conducted along the specimen. For this particular case study, the calibrated area was exclusively subjected to control (4–6, as depicted in Figure 4). The roundness (RON) was quantified as the average outcome within the measured section, based on six measurements distributed along the area. Straightness (STR) was scrutinized via four measurement lines, each separated by 90°, with each measurement line spanning a vertical length of 20 mm. CYL was then computed from these collected data.

### 2.2. Fatigue Test

**Figure 5:**
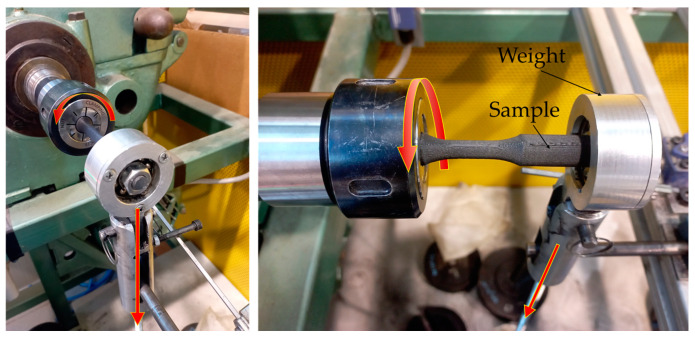
Rotational bending fatigue equipment. Sample undergoing the test.

Following the diameter measurement of the samples, the fatigue testing phase was initiated. The specimens were subjected to rotational bending fatigue testing using specialized equipment, as depicted in Figure 5. This equipment was custom-designed and fabricated at the University of Malaga. Notably, it was developed by repurposing the kinematic chain of a decommissioned parallel lathe [42,46]. The equipment’s performance and reliability have been affirmed through prior studies [30,44,47,48], thereby validating its capability to apply a single-point load to the specimen’s extremity.

In the context of the applied loads (1 and 1.5 kgf), the force was directed perpendicularly to the rotational axis, while the sample rotated at a rate of 2800 revolutions per minute. This applied load induced a sinusoidal deformation pattern within the fibers of the specimen. When the samples ultimately failed, typically occurring in the critical section subjected to the greatest bending moment, they dropped, activating a stop sensor. At this point, data regarding the number of cycles and the distance from the breaking point to the location of the load were recorded. Cycle counts were electronically tallied, while distance measurements from the breaking point were taken using a Vernier caliper with an error margin of e = 0.01 mm. This method accounts for variations in the stress experienced by different samples tested under the same weight conditions [30,38]. It is important to note that the presence of printing imperfections can accelerate crack propagation and is considered an anomalous behavior in the testing process, yet it is also acknowledged that a degree of uncertainty is inherent in fatigue studies.

Taking into consideration the specimen’s geometry and the applied load, the equations required for calculating the bending stress applied to the expected fracture section are as follows:

(1) $$S=\frac{32\cdot F\cdot \left(L-x\right)}{\pi \cdot d^{3}}$$

where the variables correspond to:

- *S*: stress at the fracture section (MPa).
- *L*: distance between the load applied section and the fixed point (mm).
- *x*: distance between the fixed point and the maximum stress point (mm).
- *d*: calibrated area diameter (mm).
- *F*: Load applied (N).

**Figure 6:**
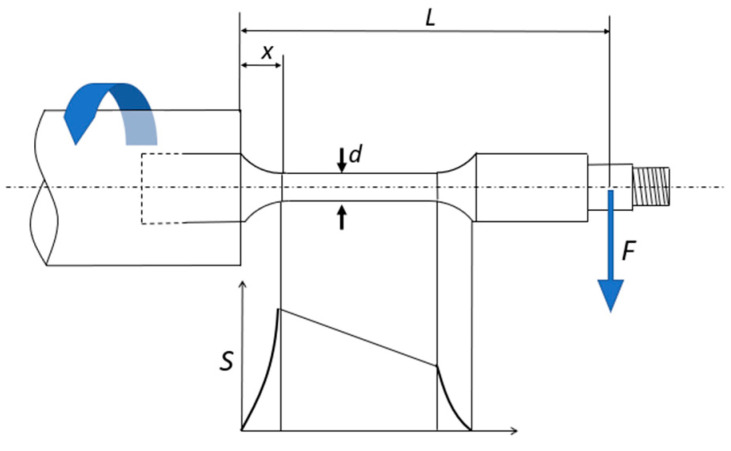
Fatigue test and stress distribution.

Figure 6 provides a visual representation of the variables essential for calculating the stress in the fracture section. It is important to note that the fatigue fracture is anticipated to manifest at the termination of the calibrated zone, which aligns with the point of maximum stress.

## 3. Results

Following the completion of fatigue testing and the measurement of the tested samples, the obtained results were subjected to comprehensive analysis to discern the impact of the various selected reinforcements.

### 3.1. Dimensional and Geometrical Results

**Figure 7:**
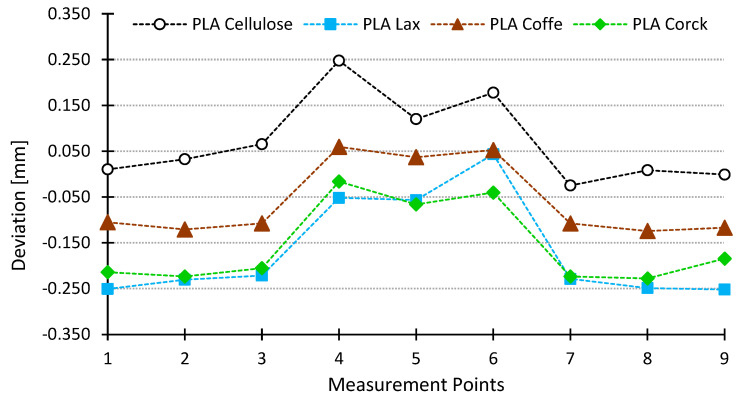
Dimensional deviations PLA + natural fibers samples.

All the samples were printed under the same printing parameters (Table 2) and conditions. Figure 7 shows the deviations obtained for the samples reinforced with natural fibers. It can be seen how samples with flax, coffee, and cork tend to offer smaller dimensions, as the deviations are negative. The center of the specimens, where the breakage occurs, is closer to the nominal measurements, with the deviations being more evident in the extreme parts of the specimen (measurement points 1–3 and 7–9). The PLA + Cellulose samples present dimensions larger than nominal, with this being more obvious for the center area (measurement points 4, 5, and 6). However, all the materials tested have similar dimensional deviations. There is higher shrinkage at the ends of the specimens (measurement points 1–3 and 7–9), corresponding to a larger diameter and therefore more material deposited.

**Figure 8:**
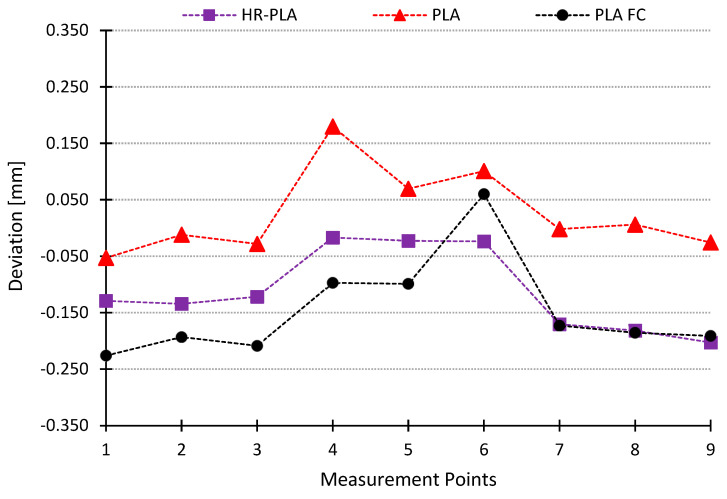
Dimensional deviations PLA + CF, HR-PLA and PLA.

A similar behavior can be seen in Figure 8, where the dimensional deviations for PLA, HR-PLA, and PLA + FC are shown. It can be established that the dimensional accuracy of the printed samples depends on the type of reinforcement and the diameter selected. The closest to the PLA behavior is the PLA + Cellulose. This behavior can be explained because the specimens that show a higher dimensional deviation compared to PLA specimens are composed of fibers with higher density. These fibers hold the temperature more, so cooling takes longer and therefore the material has more time to shrink. On the other hand, cellulose fibers, being less dense, hold less heat and cooling takes place in a shorter time, similar to the cooling of PLA-only specimens.

This behavior is complemented by the amount of material being extruded. Therefore, in the center of the part (measuring points 4–6), as there is less material, cooling occurs earlier than at the ends of the part (measuring points 1–3 and 7–9), with fewer deviations due to the lower shrinkage produced.

Furthermore, it must be taken into consideration that the additive manufacturing process using ME presents high deviations compared to other conventional manufacturing processes like machining [49,50,51].

It is clear that the manufacturing process alone makes it difficult to maintain good dimensional control. The different layers or sections of the part are cooled in different ways and at different speeds, which means that the internal stresses cause each layer to distort, giving rise to what is known as warping or shrinkage. When an area or layer cools, it shrinks. This contraction pulls on the surrounding material, which causes the phenomenon of warping or shrinkage, due to the internal tension [52]. Parts with large flat surfaces or smaller thicknesses have greater deformation.

The dimensional and geometrical deviations observed in this study can be attributed to several specific characteristics of the natural fibers used as reinforcements in PLA. The density and thermal properties of the fibers significantly affect the cooling rate during the printing process. For example, cellulose fibers, having a lower density, result in faster cooling and greater dimensional expansion, as seen with deviations up to +0.247 mm. In contrast, denser fibers like flax exhibit negative deviations up to −0.250 mm due to slower cooling and shrinkage. Additionally, the hygroscopic nature of fibers such as flax and coffee leads to moisture absorption, causing expansions or contractions during printing and cooling, further contributing to dimensional changes.

Moreover, fiber–matrix adhesion plays a crucial role in dimensional stability. Stronger interfacial bonding, as observed in cellulose-reinforced PLA, results in less warping and shrinkage, with roundness deviations around 0.01 mm compared to 0.1 mm for flax and 0.08 mm for coffee. The superior dimensional stability of cellulose-reinforced PLA is also reflected in its lower cylindricity deviation (approximately 0.05 mm), compared to higher values for flax (0.15 mm) and coffee (0.12 mm). These findings highlight the importance of fiber characteristics in determining the dimensional and geometrical outcomes of printed PLA composites.

**Figure 9:**
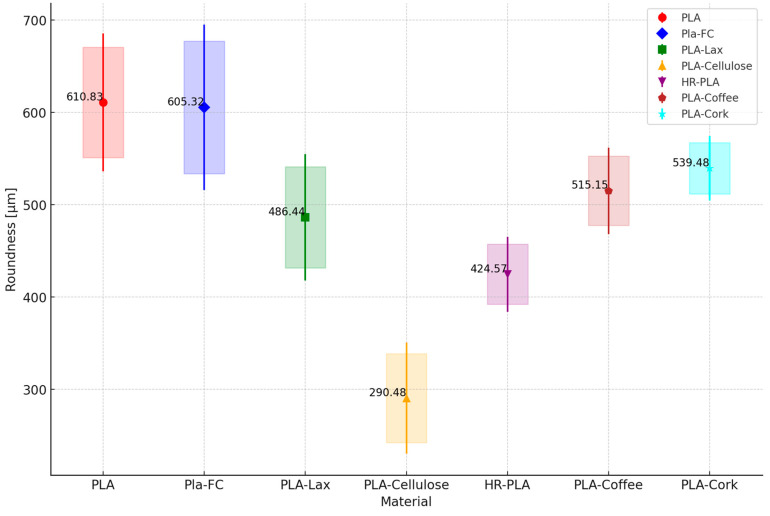
Mean, standard deviation, and confidence interval of roundness measurement.

**Figure 10:**
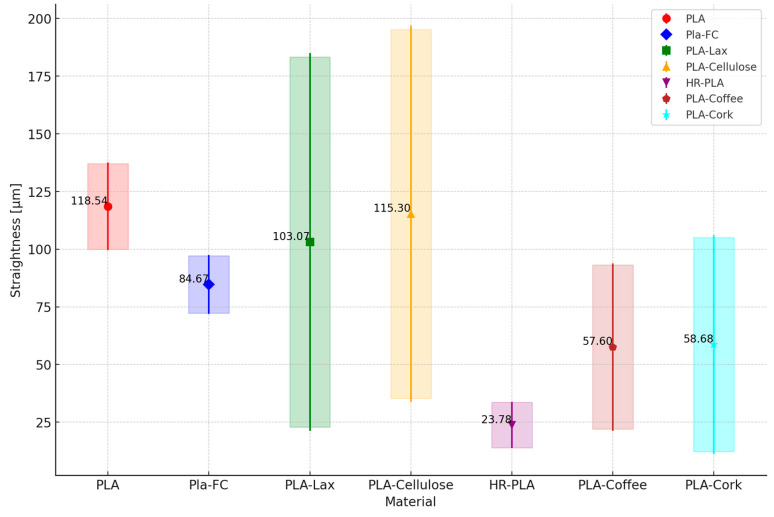
Mean, standard deviation, and confidence interval of straightness measurement.

**Figure 11:**
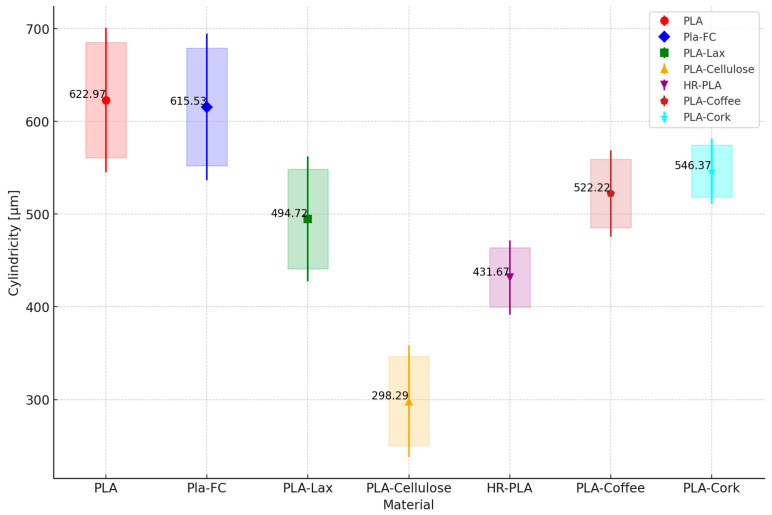
Mean, standard deviation, and confidence interval of cylindricity measurement.

As for the geometrical deviations, the results for the RON, STL, and CYL are shown in Figure 9, Figure 10 and Figure 11, respectively. Each point represents the mean straightness of the corresponding material. The vertical lines represent the standard deviations. The shaded areas represent the confidence intervals.

It can be seen that PLA and PLA + CF are the samples with the higher RON deviation, versus the PLA + Cellulose samples, which present lower RON deviation. The same behavior is shown for the CYL deviations. As for STR, PLA + Cellulose overcomes the other samples with natural reinforcement and the HR-PLA but is still lower than the PLA and PLA + CF.

With the analysis of these results, it can be concluded that natural fiber reinforcement tends to improve the geometrical deviations of the samples printed. A closer analysis of natural reinforcement could be interesting for a particular part, depending on its dimensions, geometry, and tolerances. However, it is clear that the addition of natural reinforcement will offer printed parts with fewer deviations than working with PLA or PLA + CF.

### 3.2. Fatigue Results

**Figure 12:**
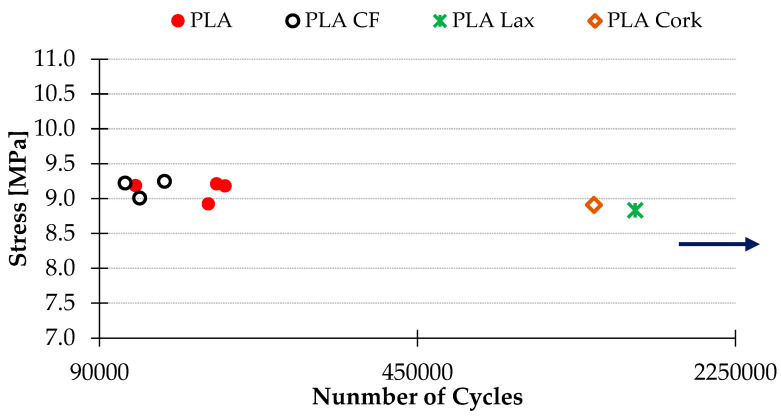
Fatigue results for materials tested with 1 kgf.

**Figure 13:**
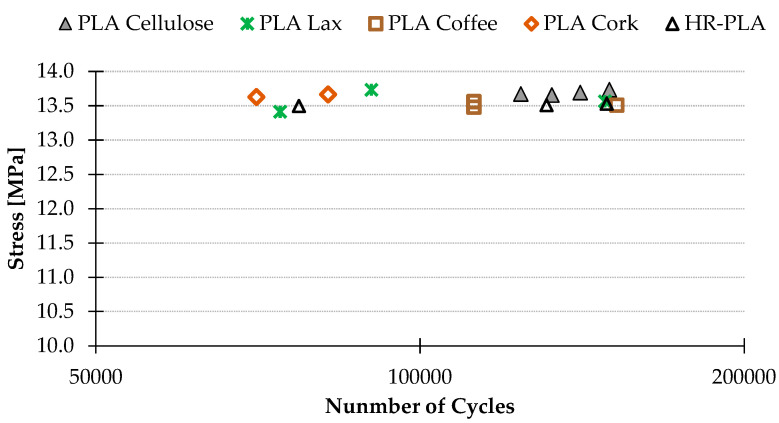
Fatigue results for materials tested with 1.5 kgf.

Loads of 1 and 1.5 kgf were tested, due to the endurance shown by several PLA + reinforcements. The natural reinforcement samples showed an infinite fatigue life for the first samples tested with 1 kgf (Figure 12), establishing the infinite fatigue life > one million cycles [42,53]. Thus, in order to determine the behavior with natural fibers, the load is increased to 1.5 kgf (Figure 13). According to this, the results showed that the implementation of natural fiber increases the load-bearing capacity of the printed material (Figure 12 and Figure 13). The PLA + Cellulose samples, for example, present a similar number of cycles as the PLA samples but with 0.5 kgf more. Figure 13 shows the behavior of the samples with natural fiber. It can be seen that the samples with PLA + CF offer the worst results. It can be appreciated that this contradicts the established general trend that carbon fiber improves the mechanical properties of a material. Correspondingly, it could be considered that as the loading is perpendicular to the direction of deposition of the material, the effect that short carbon fiber shows is less relevant in improving fatigue behavior.

The cyclic loading conditions used in this study were chosen to represent the moderate- to high-stress conditions that PLA composites might encounter in various practical applications. While the exact load levels and cycle counts were based on previous research and the available literature, different applications may experience a range of stress conditions. Our goal was to provide a preliminary understanding of the fatigue behavior of PLA composites reinforced with natural fibers under these conditions.

The fatigue tests demonstrated significant improvements in fatigue life for the reinforced PLA composites, suggesting their potential suitability for demanding environments. However, further studies are needed to fully correlate these findings with specific real-world applications. These insights provide a basis for future research to explore the performance of these materials under various stress conditions.

**Figure 14:**
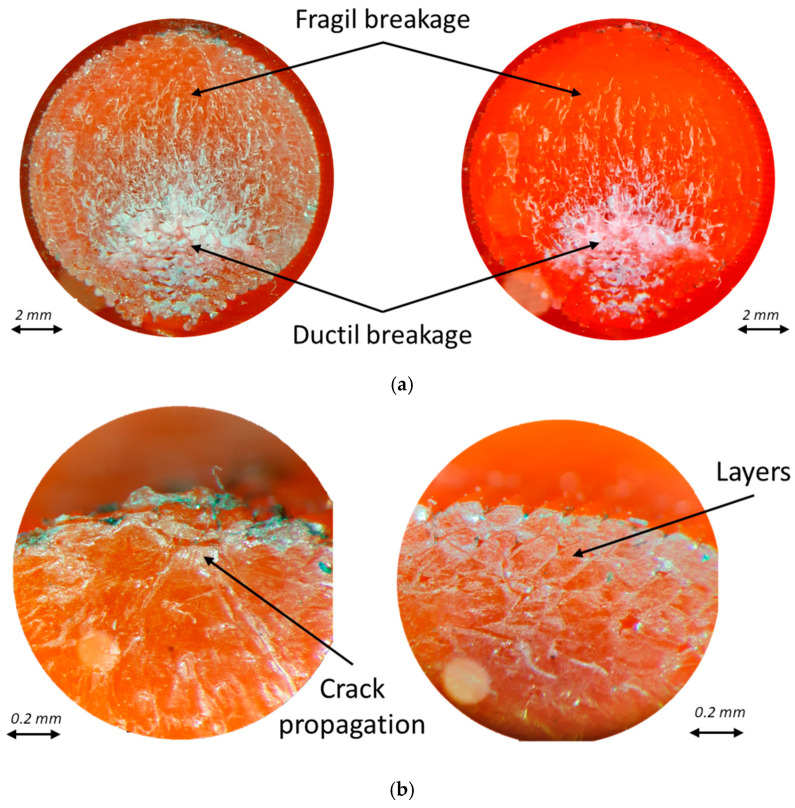
Fractography (**a**) and detail (**b**) of PLA samples.

The first fractography that can be appreciated is that of the PLA samples (Figure 14). The fracture area presents a brittle and ductile zone (Figure 14a), which can be observed in all the specimens tested. Also, good adherence between the layers and crack propagation can be appreciated (Figure 14b).

**Figure 15:**
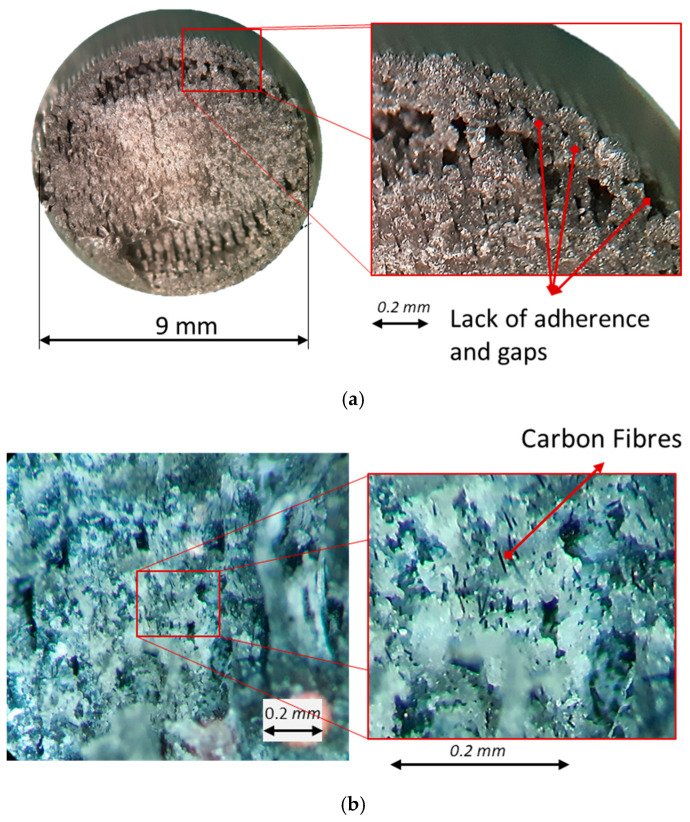
Fractography (**a**) and detail (**b**) of PLA + CF samples.

The main problem that was found with the PLA + CF specimens, compared with the PLA samples, is the lack of layer adherence and the lack of adherence between the short carbon fibers and the PLA matrix (Figure 15a). This lack of adhesion results in gaps within the specimen, which act as a stress concentrator and promote the appearance of the crack. In a comparison between the specimens with CF and PLA alone (Figure 14 and Figure 15), it can be seen that there are no gaps in the PLA specimens, presenting a more homogeneous interior. Also, the CF samples present discontinuity in the adhesion between the fiber itself and the PLA matrix, which also acts as a stress concentrator (Figure 15b).

The fracture behavior of the materials tested is similar, presenting brittle and ductile fracture zones. Therefore, the PLA base has the same behavior, with the reinforcement selection providing the difference in the number of cycles.

**Figure 16:**
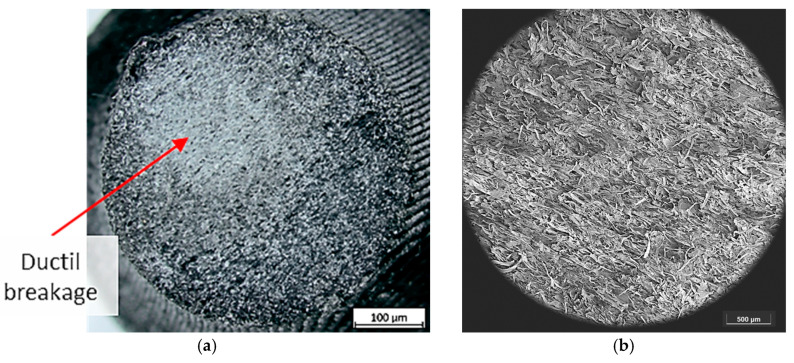

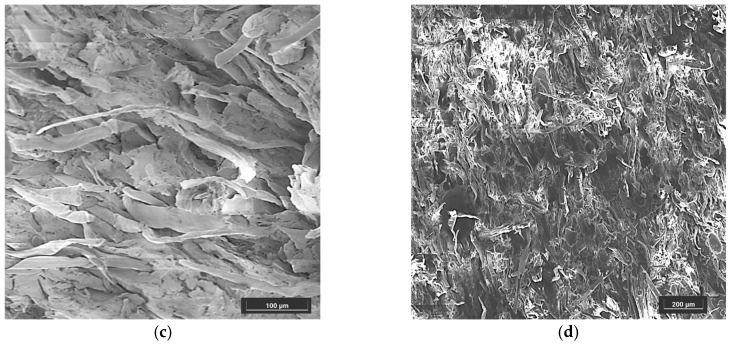
Fractography (**a**), SEM (**b**), and details (**c**,**d**) of PLA + Cellulose samples.

It can be seen that the specimens with cellulose (Figure 16) also present gaps (darker areas) in the fracture section. However, the cellulose reinforcement blends so well with the PLA matrix that it overcomes this presence of pores and offers a higher fracture toughness. Therefore, by improving the printing process and eliminating these pores, the potential of this material could be much greater.

**Figure 17:**
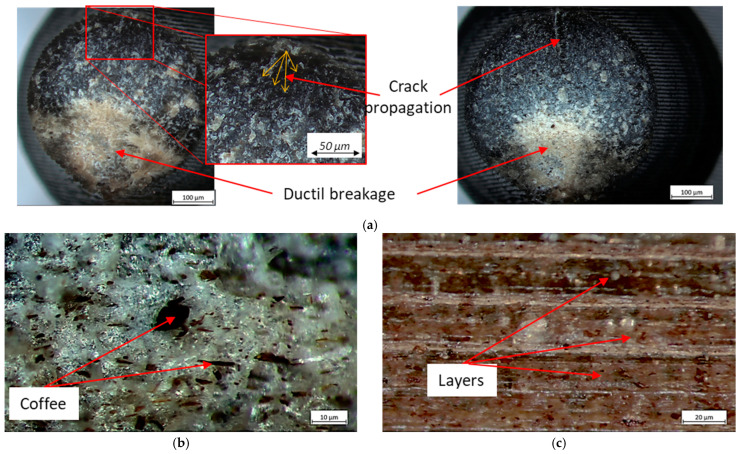

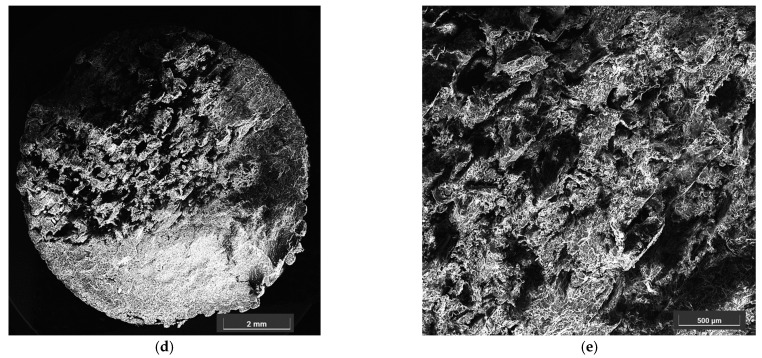
Fractography (**a**) detail (**b**,**c**), and SEM (**d**,**e**) of PLA + Coffee.

Even in the coffee specimens, where some trapped air bubbles can be seen (Figure 17), there is good interlayer adhesion and good adhesion between the coffee fibers and PLA, which also outperforms carbon fiber reinforcement and improves PLA performance.

Also, correlating the fatigue results with the geometrical deviations, it can be appreciated that the materials that offer higher fatigue resistance are also the ones that presented less geometrical deviation. So, in this case study, it can be highlighted that the natural fiber helps with the adhesion of the printed layers and also provides printed samples with fewer geometrical deviations, characteristics that improve the fatigue life of the tested samples.

In addition, it is important to highlight that the sustainability of natural fibers is a key focus of this study, as these materials offer significant environmental benefits compared to synthetic fibers. Natural fibers such as cellulose, flax, and coffee are biodegradable, reducing the environmental impact at the end of their life cycle. Studies have shown that natural fiber composites, such as those made with jute, flax, and hemp, have a lower environmental impact and carbon footprint compared to glass and carbon fiber composites. Natural fibers are renewable and have lower embodied energy compared to synthetic fibers [53,54].

Specifically, incorporating 30% by weight of natural fibers into the polypropylene (PP) matrix can reduce its carbon footprint by 3% to 18% compared to unmodified PP and PP composites with glass fibers. Moreover, life cycle assessments indicate that natural fiber composites are likely environmentally superior due to lower environmental impacts during fiber production, higher fiber content reducing more polluting base polymer content, and improved fuel efficiency during the use phase, especially in automotive applications [55].

Finally, to address the presence of significant internal gaps and voids observed in the printed specimens, which could potentially affect the reliability of the fatigue test results, we recognize the need for improved quality control measures in the fabrication process. These voids are likely the result of suboptimal printing parameters, such as an insufficient extrusion temperature or incorrect layer adhesion. Future work will focus on optimizing these parameters, including increasing the extrusion temperature and adjusting the printing speed to enhance layer bonding and reduce internal defects. Additionally, implementing a post-processing technique such as chemical smoothing or annealing could further minimize voids and improve the overall structural integrity of the printed parts.

## 4. Conclusions

In this particular case study, the enhancement of PLA through the incorporation of diverse natural reinforcements under rotational fatigue testing has been examined. The results gathered from this investigation yield several conclusions:

1. By analyzing the dimensional deviations prior to the fatigue tests, it can be seen that these deviations not only depend on the size or diameter of the part. The reinforcement introduced also has a great influence, due to its density, its capacity to dissipate heat, or its own shrinkage capacity. In this respect, the most similar behavior to the PLA specimens is those reinforced with cellulose fibers.
2. The geometrical deviations of the natural fiber reinforcement samples are lower than the samples without reinforcement or with carbon fiber. This can help when working with parts with tighter tolerances and dimensions. Also, lower geometrical deviations also improve fatigue resistance.
3. In terms of the behavior under rotational fatigue conditions, natural fibers have a much higher potential compared to carbon fibers, improving the performance of PLA by 50%, with a similar breakage mechanism, presenting brittle and ductile breakage zones.
4. Among the fibers tested, coffee and cellulose fibers performed best, with the cellulose fibers showing the best resistance. However, reduced homogeneity is observed in the cellulose specimens, with many gaps in the interior. This leads us to consider the potential of the material, improving the printing of the pieces by eliminating these voids.
5. Cork and lax fibers do not present the same improvement for the higher load (1.5 kgf), but they overcome the carbon fibers under the same load (1 kgf), improving the PLA behavior.

Practical applications: The enhanced fatigue resistance and dimensional stability of PLA composites reinforced with natural fibers, as demonstrated in this study, can have significant practical applications across various industries. For example, the aerospace and automotive sectors can benefit from these composites for non-structural and semi-structural applications, such as interior panels, trim components, and brackets, where weight reduction and sustainability are crucial. Furthermore, the use of natural fiber-reinforced PLA in consumer goods can improve the durability and lifespan of products such as sports equipment, furniture, and household items, ensuring they can withstand repeated use without significant degradation in performance. In the context of environmental sustainability, these composites offer eco-friendly packaging solutions that reduce the reliance on non-biodegradable synthetic fibers, minimizing environmental pollution and promoting the use of renewable resources.

The sustainability of natural fiber-reinforced PLA extends beyond its mechanical performance. In real-world applications, these composites offer significant environmental advantages due to their biodegradability and lower carbon footprint compared to traditional synthetic fiber composites. By reducing the reliance on non-biodegradable materials, these natural fiber composites contribute to a circular economy, particularly in industries like the automotive and aerospace sectors, where reducing the environmental impact is becoming increasingly critical. Future studies should also consider conducting life cycle assessments (LCAs) to quantify the environmental benefits of these materials in specific applications.

Limitations and Future Work: One limitation of our study is the reliance on a specific 3D-printer model (Raise 3D E2) and a set of printing parameters, which may affect the generalizability of the results. Future research should explore the impact of different printing technologies and parameter variations on the fatigue behavior of PLA composites. Additionally, the presence of internal gaps in the reinforced samples suggests the need for further optimization of the printing process to enhance material homogeneity. Investigating the long-term environmental effects and biodegradability of natural fiber-reinforced PLA in real-world applications is also recommended.

Implications for Researchers: Our findings indicate that natural fiber reinforcements can significantly enhance the mechanical properties and fatigue resistance of PLA components, making them suitable for more demanding applications in industries such as the aerospace and automotive sectors. Researchers are encouraged to explore the use of other natural fibers and hybrid composites to further improve the performance and sustainability of 3D-printed materials. The relationship between printing parameters, material properties, and fatigue performance should be a focus of future studies to develop optimized solutions for additive manufacturing.

## Figures and Tables

**Table 1:** Filament materials tested.

Material PLA +	Reinforcement Percentage [%]	Brand
Cellulose	10–20	Addnorth
Flax	10–20	Nanovia
Coffee	<10	3D Fuel
Cork	10–20	ColorFabb
HR-PLA	-	NatureWorks
CF	<15	Fillamentum
Normal	-	Smartfill

**Table 2:** Printing parameters.

Layer Thickness e [mm]	Temperature T [°C]	Speed v [mm/s]	Filling [%]	Filling Pattern	Bed Temperature T [°C]	Shells
0.2	215	40	100	Grid	55	2

## Data Availability

The original contributions presented in the study are included in the article, further inquiries can be directed to the corresponding author/s.

## References

[1] Zhao Y., Chen Y., Zhou Y. (2019). Novel Mechanical Models of Tensile Strength and Elastic Property of FDM AM PLA Materials: Experimental and Theoretical Analyses. Mater. Des..

[2] Singh S., Ramakrishna S., Singh R. (2017). Material Issues in Additive Manufacturing: A Review. J. Manuf. Process.

[3] Caminero M.Á., Chacón J.M., García-Plaza E., Núñez P.J., Reverte J.M., Becar J.P. (2019). Additive Manufacturing of PLA-Based Composites Using Fused Filament Fabrication: Effect of Graphene Nanoplatelet Reinforcement on Mechanical Properties, Dimensional Accuracy and Texture. Polymers.

[4] Wickramasinghe S., Do T., Tran P. (2020). FDM-Based 3D Printing of Polymer and Associated Composite: A Review on Mechanical Properties, Defects and Treatments. Polymers.

[5] Morales A.P., Güemes A., Fernandez-Lopez A., Valero V.C., de La Rosa Llano S. (2017). Bamboo-Polylactic Acid (PLA) Composite Material for Structural Applications. Materials.

[6] Salmi M. (2021). Additive Manufacturing Processes in Medical Applications. Materials.

[7] Mallikarjuna B., Bhargav P., Hiremath S., Jayachristiyan K.G., Jayanth N. (2023). A Review on the Melt Extrusion-Based Fused Deposition Modeling (FDM): Background, Materials, Process Parameters and Military Applications. Int. J. Interact. Des. Manuf. (IJIDeM).

[8] Osejos J.V.M., Sarria C.A., Zurita D.B.P., Rosero S.G., Jimenez G.A.M. (2019). Mechanical Capabilities of Semi-Rigid Thermoplastics ABS and PLA from 3D Printing. Int. J. Mater. Prod. Technol..

[9] Sandanamsamy L., Harun W.S.W., Ishak I., Romlay F.R.M., Kadirgama K., Ramasamy D., Idris S.R.A., Tsumori F. (2022). A Comprehensive Review on Fused Deposition Modelling of Polylactic Acid. Prog. Addit. Manuf..

[10] Chacón J.M., Caminero M.A., García-Plaza E., Núñez P.J. (2017). Additive Manufacturing of PLA Structures Using Fused Deposition Modelling: Effect of Process Parameters on Mechanical Properties and Their Optimal Selection. Mater. Des..

[11] Muthe L.P., Pickering K., Gauss C. (2022). A Review of 3D/4D Printing of Poly-Lactic Acid Composites with Bio-Derived Reinforcements. Compos. Part C Open Access.

[12] Huang H., Liu W., Liu Z. (2020). An Additive Manufacturing-Based Approach for Carbon Fiber Reinforced Polymer Recycling. CIRP Ann..

[13] Faidallah R.F., Hanon M.M., Szakál Z., Oldal I. (2024). Mechanical Characterization of 3D-Printed Carbon Fiber-Reinforced Polymer Composites and Pure Polymers: Tensile and Compressive Behavior Analysis. Int. Rev. Appl. Sci. Eng..

[14] Pertuz A.D., Díaz-Cardona S., González-Estrada O.A. (2020). Static and Fatigue Behaviour of Continuous Fibre Reinforced Thermoplastic Composites Manufactured by Fused Deposition Modelling Technique. Int. J. Fatigue.

[15] Saharudin M.S., Hajnys J., Kozior T., Gogolewski D., Zmarzły P. (2021). Quality of Surface Texture and Mechanical Properties of Pla and Pa-Based Material Reinforced with Carbon Fibers Manufactured by Fdm and Cff 3d Printing Technologies. Polymers.

[16] Najmon J.C., Raeisi S., Tovar A. (2019). Review of Additive Manufacturing Technologies and Applications in the Aerospace Industry. Additive Manufacturing for the Aerospace Industry.

[17] Liu W., Huang H., Zhu L., Liu Z. (2021). Integrating Carbon Fiber Reclamation and Additive Manufacturing for Recycling CFRP Waste. Compos. B Eng..

[18] Rahimizadeh A., Kalman J., Fayazbakhsh K., Lessard L. (2019). Recycling of Fiberglass Wind Turbine Blades into Reinforced Filaments for Use in Additive Manufacturing. Compos. B Eng..

[19] Vidakis N., Petousis M., Tzounis L., Grammatikos S.A., Porfyrakis E., Maniadi A., Mountakis N. (2021). Sustainable Additive Manufacturing: Mechanical Response of Polyethylene Terephthalate Glycol over Multiple Recycling Processes. Materials.

[20] Deb D., Jafferson J.M. (2021). Natural Fibers Reinforced FDM 3D Printing Filaments. Mater. Today Proc..

[21] Subramani R., Mustafa M.A., Ghadir G.K., Al-Tmimi H.M., Alani Z.K., Rusho M.A., Haridas D., Rajan A.J., Kumar A.P. (2024). Exploring the Use of Biodegradable Polymer Materials in Sustainable 3D Printing. Appl. Chem. Eng..

[22] Travieso-Rodriguez J.A., Zandi M.D., Jerez-Mesa R., Lluma-Fuentes J. (2020). Fatigue Behavior of PLA-Wood Composite Manufactured by Fused Filament Fabrication. J. Mater. Res. Technol..

[23] Khan F., Hossain N., Hasan F., Rahman S.M.M., Khan S., Saifullah A.Z.A., Chowdhury M.A. (2024). Advances of Natural Fiber Composites in Diverse Engineering Applications—A Review. Appl. Eng. Sci..

[24] Colorado H.A., Velásquez E.I.G., Monteiro S.N. (2020). Sustainability of Additive Manufacturing: The Circular Economy of Materials and Environmental Perspectives. J. Mater. Res. Technol..

[25] Rajendran Royan N.R., Leong J.S., Chan W.N., Tan J.R., Shamsuddin Z.S.B. (2021). Current State and Challenges of Natural Fibre-Reinforced Polymer Composites as Feeder in Fdm-Based 3d Printing. Polymers.

[26] Aliotta L., Gigante V., Coltelli M.B., Cinelli P., Lazzeri A. (2019). Evaluation of Mechanical and Interfacial Properties of Bio-Composites Based on Poly(Lactic Acid) with Natural Cellulose Fibers. Int. J. Mol. Sci..

[27] Granda L.A., Espinach F.X., Tarrés Q., Méndez J.A., Delgado-Aguilar M., Mutjé P. (2016). Towards a Good Interphase between Bleached Kraft Softwood Fibers and Poly(Lactic) Acid. Compos. B Eng..

[28] Chalid M., Rahman A., Ferdian R., Nofrijon, Priyono B. (2015). On the Tensile Properties of Polylactide (PLA)/Arenga Pinnata Ijuk Fibre Composite. Macromolecular Symposia.

[29] Travieso-Rodriguez J.A., Jerez-Mesa R., Llumà J., Gomez-Gras G., Casadesus O. (2021). Comparative Study of the Flexural Properties of ABS, PLA and a PLA–Wood Composite Manufactured through Fused Filament Fabrication. Rapid Prototyp. J..

[30] Jerez-Mesa R., Travieso-Rodriguez J.A., Llumà-Fuentes J., Gomez-Gras G., Puig D. (2017). Fatigue Lifespan Study of PLA Parts Obtained by Additive Manufacturing. Procedia Manuf..

[31] Fischer M., Schöppner V. (2017). Fatigue Behavior of FDM Parts Manufactured with Ultem 9085. JOM.

[32] Lee J., Huang A. (2013). Fatigue Analysis of FDM Materials. Rapid Prototyp. J..

[33] UNE-EN ISO 527-1:2020 Plásticos. Determinación de Las Propieda. https://www.une.org/encuentra-tu-norma/busca-tu-norma/norma/?c=N0064896.

[34] Petersmann S., Spoerk M., Van De Steene W., Üçal M., Wiener J., Pinter G., Arbeiter F. (2020). Mechanical Properties of Polymeric Implant Materials Produced by Extrusion-Based Additive Manufacturing. J. Mech. Behav. Biomed. Mater..

[35] Azadi M., Dadashi A. (2022). Experimental Fatigue Dataset for Additive-Manufactured 3D-Printed Polylactic Acid Biomaterials under Fully-Reversed Rotating-Bending Bending Loadings. Data Brief..

[36] Hassanifard S., Hashemi S.M. (2020). On the Strain-Life Fatigue Parameters of Additive Manufactured Plastic Materials through Fused Filament Fabrication Process. Addit. Manuf..

[37] Parast M.S.A., Bagheri A., Kami A., Azadi M., Asghari V. (2022). Bending Fatigue Behavior of Fused Filament Fabrication 3D-Printed ABS and PLA Joints with Rotary Friction Welding. Prog. Addit. Manuf..

[38] Bagheri A., Aghareb Parast M.S., Kami A., Azadi M., Asghari V. (2022). Fatigue Testing on Rotary Friction-Welded Joints between Solid ABS and 3D-Printed PLA and ABS. Eur. J. Mech. A/Solids.

[39] (2010). Metallic Materials—Rotating Bar Bending Fatigue Testing.

[40] Bermudo Gamboa C., Martín Béjar S., Trujillo Vilches F.J., Sevilla Hurtado L. (2022). Geometrical Analysis in Material Extrusion Process with Polylactic Acid (PLA)+carbon Fiber. Rapid Prototyp. J..

[41] Martín Béjar S., Trujillo Vilches F.J., Bermudo Gamboa C., Sevilla Hurtado L. (2020). Fatigue Behavior Parametric Analysis of Dry Machined UNS A97075 Aluminum Alloy. Metals.

[42] Montesinos A.G., Gamboa C.B., Bejar S.M., Hurtado L.S. (2023). Influence of Layer Thickness on Fatigue Life of PLA+ Carbon Fiber Specimens by Additive Manufacturing.

[43] SOLIDWORKS. https://www.solidworks.com/es.

[44] Powerful 3D Slicer Software: IdeaMaker by Raise3D. https://www.raise3d.com/ideamaker/.

[45] (2003). Metallic Materials—Fatigue Testing—Statistical Planning and Analysis of Data.

[46] Trujillo F.J., Martín-Béjar S., Bermudo C., Sevilla L. (2018). Fatigue Test Bench Manufacturing by Reusing a Parallel Lathe. Advances in Manufacturing Technology XXXII.

[47] Martín Bejar S. (2020). Análisis Paramétrico Del Comportamiento a Fatiga de Piezas Torneadas En Seco de La Aleación UNS A97075 (Al-Zn). Ph.D. Thesis.

[48] Gómez-Gras G., Pérez M.A., Fábregas-Moreno J., Reyes-Pozo G. (2021). Experimental Study on the Accuracy and Surface Quality of Printed versus Machined Holes in PEI Ultem 9085 FDM Specimens. Rapid Prototyp. J..

[49] Buj-Corral I., Zayas-Figueras E.E. (2023). Comparative Study about Dimensional Accuracy and Form Errors of FFF Printed Spur Gears Using PLA and Nylon. Polym. Test..

[50] Zayas-Figueras E.E., Buj-Corral I. (2023). Comparative Study about Dimensional Accuracy and Surface Finish of Constant-Breadth Cams Manufactured by FFF and CNC Milling. Micromachines.

[51] Redwood B., Schöffer F., Garret B. (2017). The 3D Printing Handbook: Technologies, Design and Applications.

[52] Kiani P., Sedighi M., Kasaeian-Naeini M., Jabbari A.H. (2023). High cycle fatigue behavior and thermal properties of PLA/PCL blends produced by fused deposition modeling. J. Polym. Res..

[53] Korol J., Hejna A., Burchart-Korol D., Wachowicz J. (2020). Comparative Analysis of Carbon, Ecological, and Water Footprints of Polypropylene-Based Composites Filled with Cotton, Jute and Kenaf Fibers. Materials.

[54] Meredith J., Ebsworth R., Coles S.R., Wood B.M., Kirwan K. (2012). Natural Fibre Composite Energy Absorption Structures. Compos. Sci. Technol..

[55] Joshi S.V., Drzal L.T., Mohanty A.K., Arora S. (2004). Are Natural Fiber Composites Environmentally Superior to Glass Fiber Reinforced Composites?. Compos. Part. A Appl. Sci. Manuf..

